# Supplementary material for: Characterization of microRNAs and Target Genes in Musa acuminata subsp. burmannicoides, var. Calcutta 4 during Interaction with Pseudocercospora musae
Source: Plants (Basel). 2023 Mar 28;12(7):1473. doi: 10.3390/plants12071473 (PMC10097032; doi:10.3390/plants12071473)
Supplement: Supplementary file 1 [file plants-12-01473-s001.zip › SUPPLEMENTARY_FIGURE_S1.pdf]

**SUPPLEMENTARY FIGURE S1.** Predicted hairpin secondary structure of novel *Musa acuminata* var. Calcutta 4 miRNAs. Secondary structures were determined using the program Forna. Mature sequences are highlighted in light blue. Star sequences are highlighted in light green. A putative star sequence is highlighted in light grey.

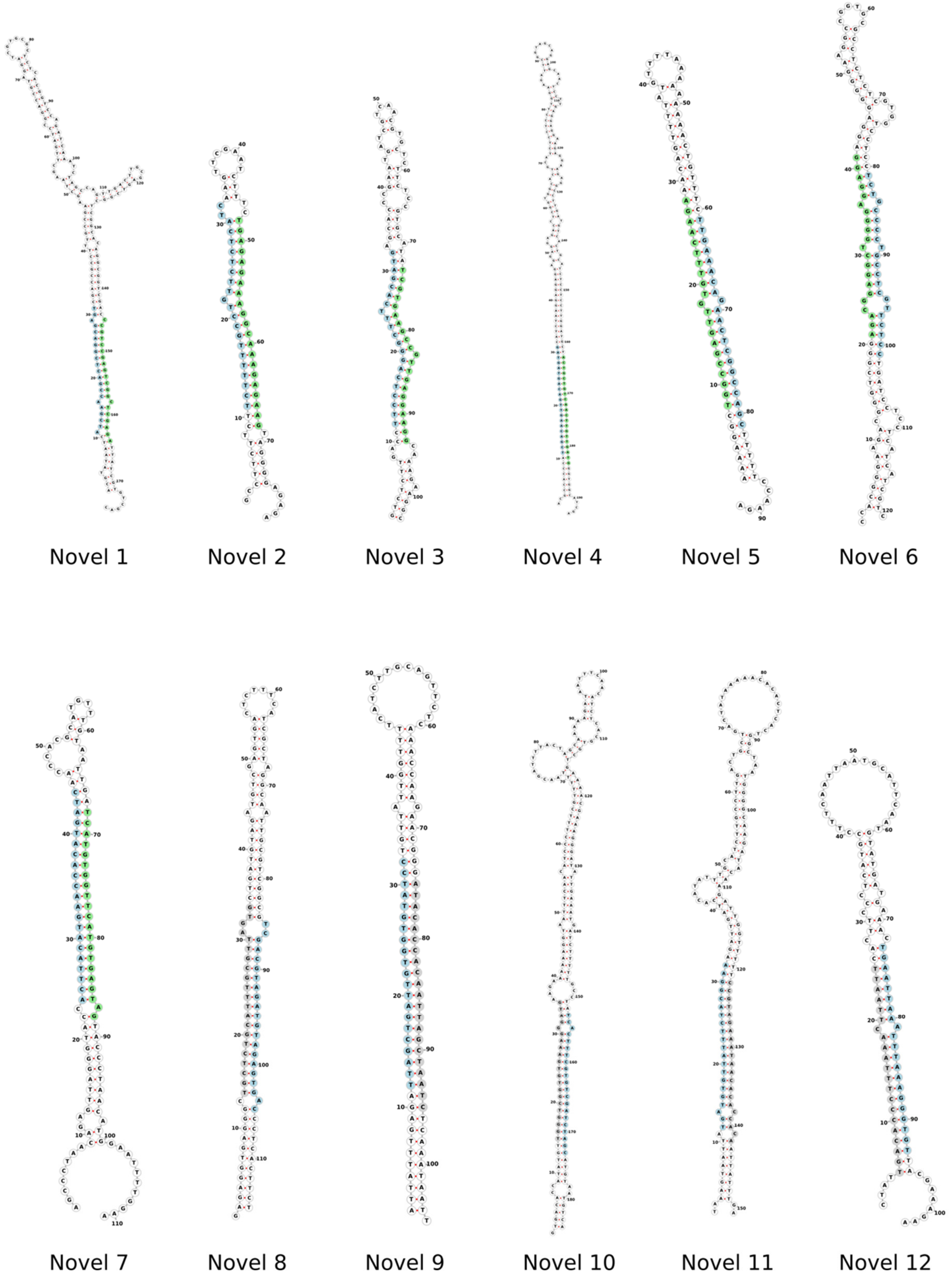

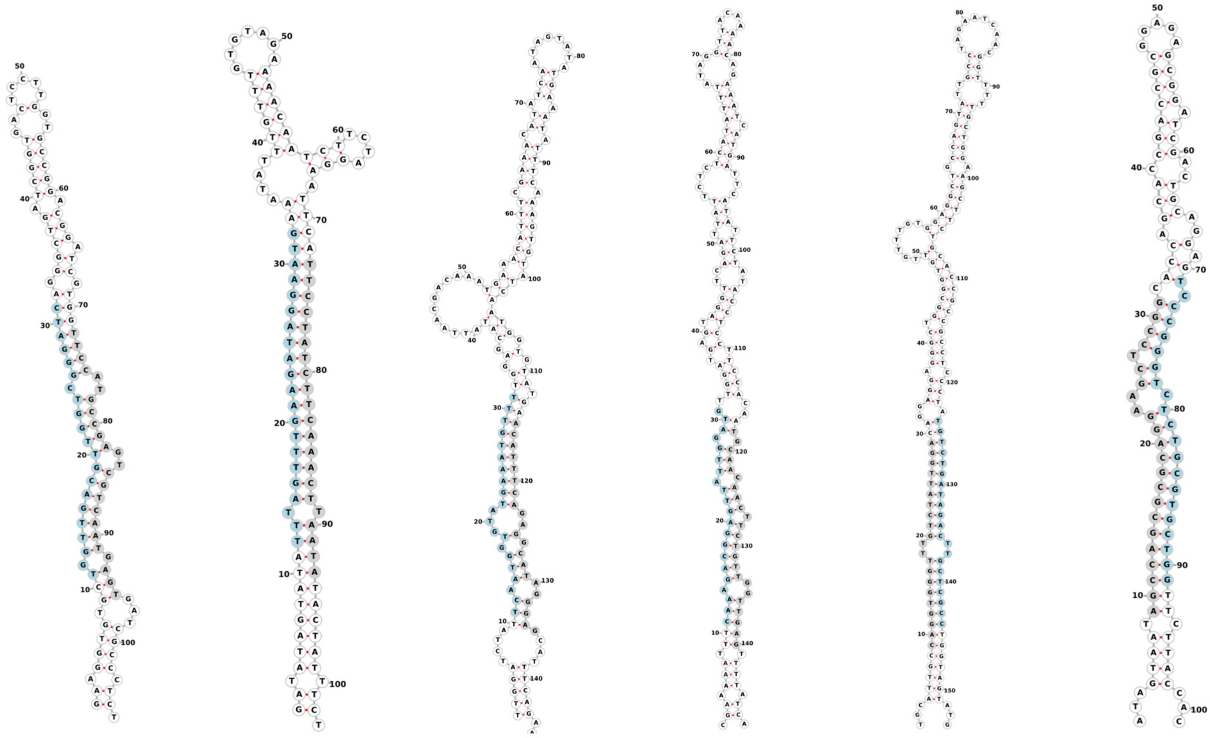

Novel 13 Novel 14 Novel 15 Novel 16 Novel 17 Novel 18

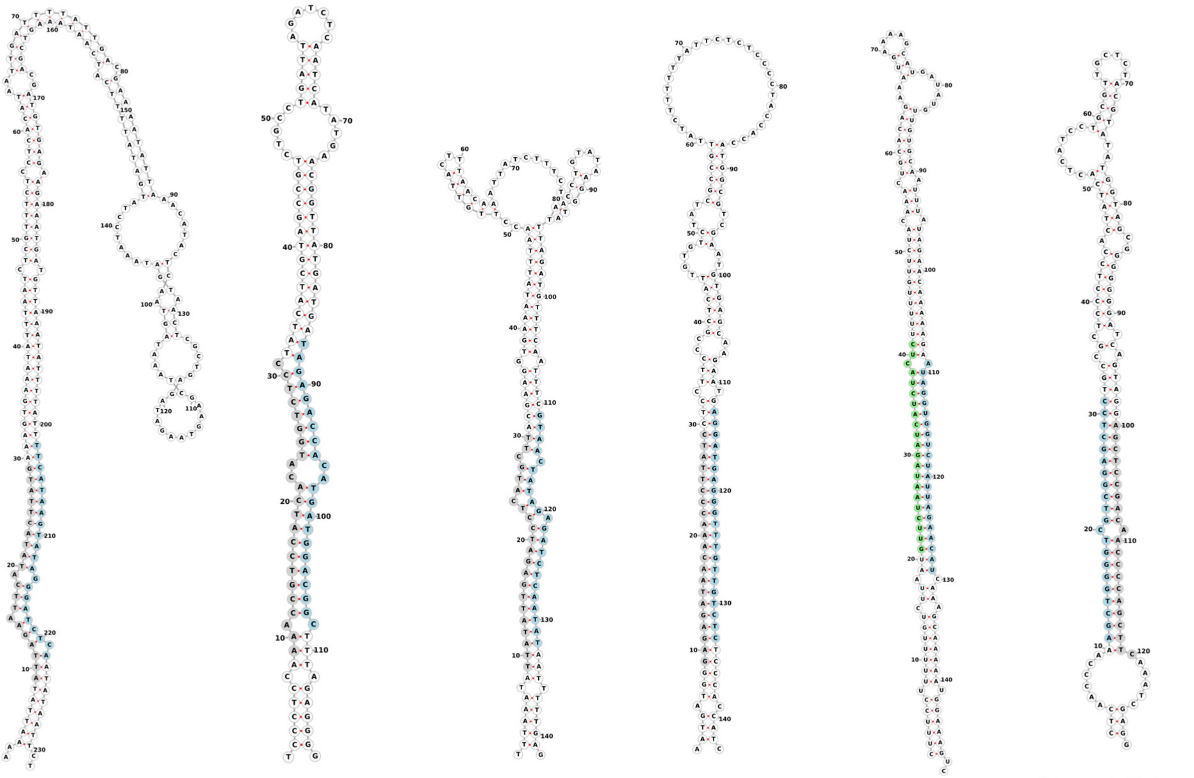

Novel 19 Novel 20 Novel 21 Novel 22 Novel 23 Novel 24
