# Supplementary material for: Characterization of microRNAs and Target Genes in Musa acuminata subsp. burmannicoides, var. Calcutta 4 during Interaction with Pseudocercospora musae
Source: Plants (Basel). 2023 Mar 28;12(7):1473. doi: 10.3390/plants12071473 (PMC10097032; doi:10.3390/plants12071473)
Supplement: Supplementary file 1 [file plants-12-01473-s001.zip › SUPPLEMENTARY_FIGURE_S2.pdf]

SUPPLEMENTARY FIGURE S2: Examples of TargetFinder-derived alignments between novel predicted *Musa acuminata* var. Calcutta 4 miRNAs and putative gene targets. Scores above 4 were not considered.

Novel #1

```
query=chr01:7155057-7155266, target=GG22|c6670_g1_i1, score=3.5, range=550-569, strand=1

Annot: gnl|BL_ORD_ID|280540     Q9FHK9.1 RecName: Full=Probable mediator of RNA polymerase II transcription subunit 26b


target  5' GCUCGUCCGGGUCGGUU-GAU 3'
           .::::::::.::::::: :::
query   3' UGAGCAGGCUCAGCCAAGCUA 5'
```

Novel #2

```
query=chr05:5132735-5132813, target=GG150|c3487_g1_i2, score=4, range=0-0, strand=1

Annot: gnl|BL_ORD_ID|159087     Q0E2Z7.2 RecName: Full=DEAD-box ATP-dependent RNA helicase 41


target  5' GGUGAGGGAACAGG-AAAAGGA 3'
           :.::::.::::::: ::::.::
query   3' CUACUCUCUUGUCCGUUUUUCU 5'
```

Novel #3

```
query=chr07:20901036-20901267, target=GG52|c3151_g1_i1, score=3.5, range=639-658, strand=1

Annot: gnl|BL_ORD_ID|18692      P19182.2 RecName: Full=Interferon-related developmental regulator 1; AltName: Full=Nerve growth factor-inducible protein PC4; AltName: Full=T
PA-induced sequence 7; Short=TIS7 protein


target  5' CAU-GUUGAGGCCCUGAGGAA 3'
           ::: :: .:.:::::::::::
query   3' GUAGCACUUUCGGGACUCCUU 5'
```

Novel #4

```
query=chr08:3639196-3639285, target=GG22|c3772_g1_i1, score=0, range=1629-1649, strand=1

Annot: gnl|BL_ORD_ID|24032      Q8NHP7.4 RecName: Full=piRNA biogenesis protein EXD1; AltName: Full=Exonuclease 3&apos;-5&apos; domain-containing protein 1; AltName: Full=Ex
onuclease 3&apos;-5&apos; domain-like-containing protein 1; AltName: Full=Inactive exonuclease EXD1


target  5' CUACUACUGUCAACUUGUCGA 3'
           :::::::::::::::::::::
query   3' GAUGAUGACAGUUGAACAGCU 5'
```

Novel #5

```
query=chr10:34402590-34402681, target=GG52|c2643_g2_i4, score=3.5, range=1260-1280, strand=1

Annot: gnl|BL_ORD_ID|280703     Q9CAG8.1 RecName: Full=F-box/kelch-repeat protein At1g67480


target  5' GCGUGAGACAGAACUUGGCCA 3'
             : :::.::::::::.:::::
query   3' AGAACUUUGUCUUGAGCCGGU 5'
```

Novel #6

```
query=chr09:11246782-11246863, target=GG22|c2238_g2_i1, score=3, range=3074-3095, strand=1

Annot: gnl|BL_ORD_ID|272462     Q9FN92.1 RecName: Full=Probable receptor-like protein kinase At5g59700; Flags: Precursor


target  5' GGGCAGCAGAGGCAGGGGCAGA 3'
           ::. :.: ::::::::::::::
query   3' CCUCUUG-CUCCGUCCCCGUCU 5'
```

Novel #7

```
query=chr09:34735640-34735819, target=GG24|c5249_g1_i2, score=4, range=924-944, strand=1

Annot: gnl|BL_ORD_ID|427076     P46547.3 RecName: Full=Proline iminopeptidase; Short=PIP; AltName: Full=Prolyl aminopeptidase; Short=PAP


target  5' GCAUUAUUCACAUGGAUCACA 3'
           : :.::.:::::::.:.::::
query   3' CAUGAUGAGUGUACUUGGUGU 5'
```

Novel#8

```
query=chr01:3237626-3237740, target=GG50|c3177_g2_i3, score=2, range=676-696, strand=1

Annot: gnl|BL_ORD_ID|370403     Q9LSG3.1 RecName: Full=Galacturonosyltransferase 8; AltName: Full=Glycosyltransferase QUASIMODO1


target  5' GUCGCUCUGCAUCUACGUCGG 3'
           :::.::::.::::::::::::.
query   3' CAGUGAGAUGUAGAUGCAGCU 5'
```

Novel #9

```
query=chr01:7762935-7763039, target=GG41|c2631_g1_i1, score=3.5, range=777-797, strand=1

Annot: gnl|BL_ORD_ID|324572     Q9SE42.1 RecName: Full=Ribulose-phosphate 3-epimerase, cytoplasmic isoform; AltName: Full=Cyt-RPEase; AltName: Full=Pentose-5-phosphate 3-epi
merase; Short=PPE; AltName: Full=RPEcyt; AltName: Full=Ribulose-5-phosphate-epimerase


target  5' GGAUAAAUCAUAAUCAGUUAA 3'
           ::::: :.::.::::::.:::
query   3' CCUAUGUGGUGUUAGUCGAUU 5'
```

Novel #10

```
query=chr02:15328566-15328749, target=GG52|c3155_g1_i1, score=3, range=630-649, strand=1

Annot: gnl|BL_ORD_ID|5152       B8B9K6.1 RecName: Full=60S ribosomal protein L10a B7F845.1 RecName: Full=60S ribosomal protein L10a


target  5' GCAAGAU-GAUACGAAAGUGA 3'
           :: :::: ::.::::::::::
query   3' CGAUCUAGCUGUGCUUUCACU 5'
```

Novel #11

```
query=chr02:22236894-22237044, target=GG40|c2316_g1_i1, score=0, range=691-711, strand=1

Annot: gnl|BL_ORD_ID|5278       K4BVL1.1 RecName: Full=Protein SPA, chloroplastic; AltName: Full=Sugar partition-affecting protein; Flags: Precursor


target  5' UUCCGUAGAAAUAACACAUCA 3'
           :::::::::::::::::::::
query   3' AAGGCAUCUUUAUUGUGUAGU 5'
```

Novel #12

```
query=chr02:27471405-27471507, target=GG50|c3293_g1_i8, score=4, range=791-810, strand=1

Annot: gnl|BL_ORD_ID|359984     Q94KB2.1 RecName: Full=MLO-like protein 13; Short=AtMlo13; Short=AtMlo20


target  5' AU-CUCUUUAAACUUAAUUCA 3'
           :. :.::::::: ::::::::
query   3' UGUGGGAAAUUUAAAUUAAGU 5'
```

Novel #13

```
query=chr04:10607231-10607336, target=GG98|c9128_g1_i1, score=2.5, range=140-159, strand=1

Annot: gnl|BL_ORD_ID|850        A0A140IL90.1 RecName: Full=Noroxomaritidine synthase; AltName: Full=CYP96T1; AltName: Full=Cytochrome P450 96T1


target  5' GA-CCCGGCCAACGUUAACCA 3'
           :: ::::.:::::::.:::::
query   3' CUAGGGCUGGUUGCAGUUGGU 5'
```

Novel #14

```
query=chr04:30762239-30762336, target=GG22|c4367_g1_i1, score=3, range=1930-1950, strand=1

Annot: gnl|BL_ORD_ID|194080     Q2RBN7.1 RecName: Full=Clathrin heavy chain 1


target  5' CAUGCUUUUCUUCAAACUAAG 3'
           ::: :.: ::::::::::::.
query   3' GUAAGGAUAGAAGUUUGAUUU 5'
```

Novel $15

```
query=chr05:22232500-22232644, target=GG52|c3654_g5_i2, score=4, range=1037-1056, strand=1

Annot: gnl|BL_ORD_ID|210607     Q7XA42.2 RecName: Full=Putative disease resistance protein RGA1; AltName: Full=RGA3-blb


target  5' AAGAUUUUGUGCACCAUUGA 3'
           :: ::::..:.:::::::::
query   3' UUGUAAAGUAUGUGGUAACU 5'
```

Novel #16

```
query=chr05:25109802-25109949, target=GG52|c3471_g1_i1, score=0, range=3077-3097, strand=1

Annot: gnl|BL_ORD_ID|273094     Q8GSA7.1 RecName: Full=Calmodulin-binding transcription activator 3; AltName: Full=Ethylene-induced calmodulin-binding protein 1; Short=EICBP
1; AltName: Full=Ethylene-induced calmodulin-binding protein a; Short=EICBP.a; AltName: Full=Signal-responsive protein 1


target  5' CAUCCAAUAACUCCGUCUUUG 3'
           :::::::::::::::::::::
query   3' GUAGGUUAUUGAGGCAGAAAC 5'
```

Novel #17

```
query=chr08:39270564-39270717, target=GG22|c7255_g1_i1, score=0, range=2025-2045, strand=1

Annot: gnl|BL_ORD_ID|371080     O80822.2 RecName: Full=Peroxidase 25; Short=Atperox P25; Flags: Precursor


target  5' GGCGAGCAAGUCUAUCAGACA 3'
           :::::::::::::::::::::
query   3' CCGCUCGUUCAGAUAGUCUGU 5'
```

Novel #18

```
query=chr09:34029225-34029338, target=GG15|c3059_g1_i1, score=3, range=672-692, strand=1

Annot: gnl|BL_ORD_ID|282855     Q6AST1.1 RecName: Full=Homeobox-leucine zipper protein HOX32; AltName: Full=HD-ZIP protein HOX32; AltName: Full=Homeodomain transcription fac
tor HOX32; AltName: Full=OsHox32


target  5' UUGGGAUGAAGCCUGGUCCGG 3'
             ::.:::::::::::::::.
query   3' CCCCUUACUUCGGACCAGGCU 5'
```

Novel #19

```
query=chr09:40438631-40438862, target=GG15|c2014_g1_i1, score=0.5, range=153-173, strand=1

Annot: gnl|BL_ORD_ID|271714     Q9XIB6.1 RecName: Full=Pollen-specific leucine-rich repeat extensin-like protein 2; Short=AtPEX2; Short=Pollen-specific LRR/EXTENSIN2; AltNam
e: Full=Cell wall hydroxyproline-rich glycoprotein; Flags: Precursor


target  5' UGGGAUCCCUAUACUUAUGAA 3'
           ::.::::::::::::::::::
query   3' ACUCUAGGGAUAUGAAUACUU 5'
```

Novel #20

```
query=chr10:26852029-26852146, target=GG50|c3533_g1_i1, score=3.5, range=1575-1594, strand=1

Annot: gnl|BL_ORD_ID|273933     Q9ZQ96.1 RecName: Full=UDP-glycosyltransferase 73C3


target  5' ACGUUC-UCAUGUGGUCUCUU 3'
             :::.: ::::::::::::
query   3' GGCAGGUAGUACACCAGAGAU 5'
```

Novel #21

```
query=chr11:2322440-2322581, target=GG50|c3177_g2_i1, score=3.5, range=350-371, strand=1

Annot: gnl|BL_ORD_ID|370403     Q9LSG3.1 RecName: Full=Galacturonosyltransferase 8; AltName: Full=Glycosyltransferase QUASIMODO1


target  5' AUAUUGAAAUCCCUAUAGUUAU 3'
            :::::: ::: ::::::::.
query   3' UAUAACUCUAGAGAUAUCAAUG 5'
```

Novel #22

```
query=chr11:5710224-5710366, target=GG50|c3177_g2_i1, score=0.5, range=1001-1021, strand=1

Annot: gnl|BL_ORD_ID|370403     Q9LSG3.1 RecName: Full=Galacturonosyltransferase 8; AltName: Full=Glycosyltransferase QUASIMODO1


target  5' GAGACAGCAAACCCUCAUCCU 3'
           ::::::.::::::::::::::
query   3' CUCUGUUGUUUGGGAGUAGGA 5'
```

Novel #23

```
Annot: gnl|BL_ORD_ID|11109      F4JSH1.1 RecName: Full=Probable apyrase 7; Short=AtAPY7; AltName: Full=ATP-diphosphatase; AltName: Full=ATP-diphosphohydrolase; AltName: Full
=Adenosine diphosphatase; Short=ADPase; AltName: Full=NTPDase; AltName: Full=Nucleoside triphosphate diphosphohydrolase 7


target  5' UUGGUC-AAUAGACCACUUAU 3'
            :: :: ::::::::::.:::
query   3' UACAAGAUUAUCUGGUGGAUA 5'
```

Novel #24

```
query=chr07:1353404-1353488, target=GG22|c5693_g1_i1, score=4, range=228-248, strand=1

Annot: gnl|BL_ORD_ID|278947     Q9SU13.1 RecName: Full=Fasciclin-like arabinogalactan protein 2; Flags: Precursor


target  5' GGGGCGUCGACGACCUCAGCG 3'
           ::.:: .::::::::.::::
query   3' CCUCGAGGCUGCUGGGGUCGA 5'
```
