# Supplementary material for: Characterization of microRNAs and Target Genes in Musa acuminata subsp. burmannicoides, var. Calcutta 4 during Interaction with Pseudocercospora musae
Source: Plants (Basel). 2023 Mar 28;12(7):1473. doi: 10.3390/plants12071473 (PMC10097032; doi:10.3390/plants12071473)
Supplement: Supplementary file 1 [file plants-12-01473-s001.zip › SUPPLEMENTARY_FIGURE_S3.pdf]

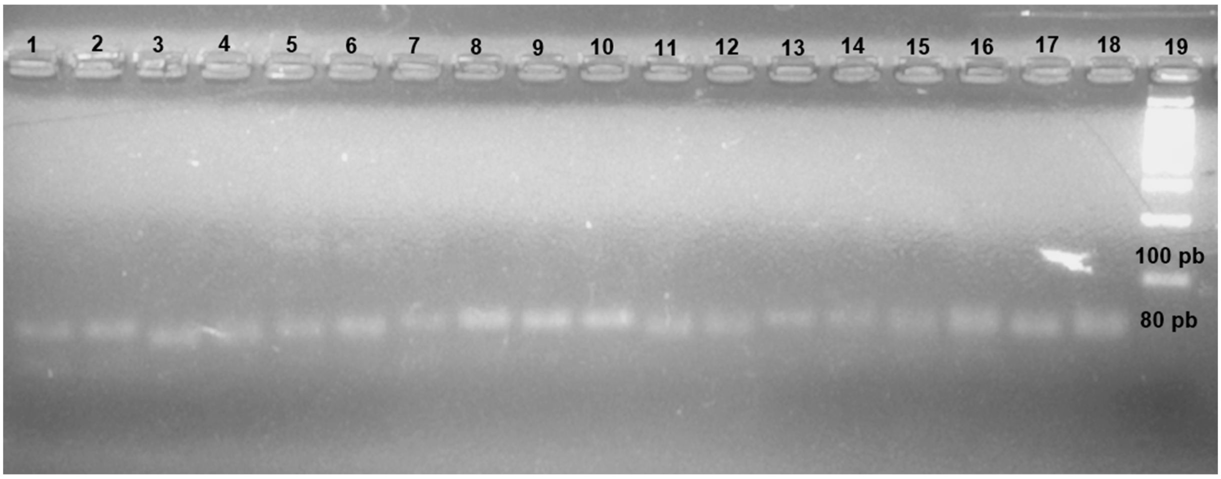

SUPPLEMENTARY FIGURE S3. RT-PCR-derived amplicons of expected product size for *Musa* miRNAs. 1: miR171a-3p_I; 2: miR171a-3p_NI; 3: miR164c-5p_I; 4: miR164c-5p_NI; 5: mi171e-3p_I; 6: mi171e-3p_NI; 7: miR393b-3p_I; 8: miR393b-3p_NI; 9: miR397a-5p_I; 10: miR397a-5p_NI; 11: miR167a-3p_I; 12: miR167a-3p_NI; 13: miR167b-3p_I; 14: miR167b-3p_NI; 15: miR167a-5p_I; 16: miR167a-5p_NI; 17: miR535d-5p_I; 18: miR535d-5p_NI; 19: 100bp DNA ladder (Promega).
