# Supplementary material for: Characterization of microRNAs and Target Genes in Musa acuminata subsp. burmannicoides, var. Calcutta 4 during Interaction with Pseudocercospora musae
Source: Plants (Basel). 2023 Mar 28;12(7):1473. doi: 10.3390/plants12071473 (PMC10097032; doi:10.3390/plants12071473)
Supplement: Supplementary file 1 [file plants-12-01473-s001.zip › SUPPLEMENTARY_TABLE_S1.pdf]

SUPPLEMENTARY TABLE S1. Summary statistics for all known conserved and novel *Musa acuminata* var. Calcutta 4 miRNAs

**Family**	**Loci**	**Paired miRNAs**	**Unpaired miRNAs**	**Unique major miRNAs**	**Unique miRNA-miRNA***
miR156	25	19	6	6	17
miR157	4	4	0	1	2
miR159	7	4	3	3	3
miR160	10	5	5	3	2
miR162	6	6	0	2	3
miR164	10	9	1	4	8
miR166	20	18	2	3	9
miR167	14	8	6	4	6
miR168	9	9	0	2	5
miR169	6	2	4	3	1
miR171	19	17	2	8	13
miR172	14	13	1	2	7
miR319	3	2	1	3	2
miR390	5	5	0	2	5
miR391	2	2	0	2	2
miR393	6	4	2	2	4
miR395	3	2	1	1	2
miR396	17	16	1	5	15
miR397	1	1	0	1	1
miR398	1	1	0	1	1
miR399	1	0	1	0	0
miR408	1	1	0	1	1
miR482	1	1	0	1	1
miR528	1	1	0	1	1
miR529	4	4	0	2	2
miR530	1	1	0	1	1
miR535	8	8	0	2	6
miR827	1	1	0	1	1
miR1432	1	1	0	1	1
miR2118	1	1	0	1	1
Novel	24	9	15	9	9
**Total**	**226**	**175**	**51**	**78**	**132**

$
