# Supplementary material for: Characterization of microRNAs and Target Genes in Musa acuminata subsp. burmannicoides, var. Calcutta 4 during Interaction with Pseudocercospora musae
Source: Plants (Basel). 2023 Mar 28;12(7):1473. doi: 10.3390/plants12071473 (PMC10097032; doi:10.3390/plants12071473)
Supplement: Supplementary file 1 [file plants-12-01473-s001.zip › SUPPLEMENTARY_TABLE_S5_CORRECTED.pdf]

Supplementary Table S5: List of specific primers for stem-loop RT-qPCR and conventional RT-qPCR validation of expression of selected *Musa acuminata* var. Calcutta 4 miRNAs and *Musa* target genes

Primer sequences and supporting information for stem-loop RT-PCR analysis of differential gene expression of *Musa* miRNAs during interaction with *Pseudocercopsora musae*			
**Musa miRNA**	**Primer**	**Primer sequence (5'-3')**	**Expected Amplicon size (bp)**
miR397a-5p	Stem-loop RT	GTTGGCTCTGGTGCAGGGTCCGAGGTATTCGCACCAGAGCCAAC TCTCAT	
	Forward	GTTGTTGAGTGCAGCGTTG	
	Universal reverse	GTGCAGGGTCCGAGGT	80 pb
miR167a-3p	Stem-loop RT	GTTGGCTCTGGTGCAGGGTCCGAGGTATTCGCACCAGAGCCAACTCAGAT	
	Forward	GTTTGAAGCTGCCAGCATG	
	Universal reverse	GTGCAGGGTCCGAGGT	80 pb
miR393b-3p	Stem-loop RT	GTTGGCTCTGGTGCAGGGTCCGAGGTATTCGCACCAGAGGCCAACGATC	
	Forward	GTTGTCCAAACGGATCCA	
	Universal reverse	GTGCAGGGTCCGAGGT	80 pb
miR171a-3p	Stem-loop RT	TTGGCTCTGGTGCAGGGTCCGAGGTATTCGCACCAGAGCCAACGATA	
	Forward	GTTTTGATTGAGCCGTGCC	
	Universal reverse	GTGCAGGGTCCGAGGT	80 pb
miR171e-3p	Stem-loop RT	GTTGGCTCTGGTGCAGGGTCCGAGGTATTCGCACCAGAGCCAACAGAGAT	
	Forward	GTTGTTGAGCCGCGTCAAT	
	Universal reverse	GTGCAGGGTCCGAGGT	80 pb
miR535d-5p	Stem-loop RT	GTTGGCTCTGGTGCAGGGTCCGAGGTATTCGCACCAGAGCCAACGCGTGC	
	Forward	GTGGTGACAACGAGAGAGA	
	Universal reverse	GTGCAGGGTCCGAGGT	80 pb
miR156b-5p	Stem-loop RT	GTTGGCTCTGGTGCAGGGTCCGAGGTATTCGCACCAGAGCCAAC GTGCTC	
	Forward	GTTGGTGACAGGAGAGAGT	80 pb
	Universal reverse	GTGCAGGGTCCGAGGT	
miR167b-3p	Stem-loop RT	GTTGGCTCTGGTGCAGGGTCCGAGGTATTCGCACCAGAGCCAAC TCAGAT	
	Forward	GTGTAAAGCTGCCAGCATG	
	Universal reverse	GTGCAGGGTCCGAGGT	80 pb
miR167a-5p	Stem-loop RT	GTTGGCTCTGGTGCAGGGTCCGAGGTATTCGCACCAGAGCCAACTCAGAT	
	Forward	GTTTCAAGCTGCCAGCATG	
	Universal reverse	GTGCAGGGTCCGAGGT	80 pb
miR164c-5p	Stem-loop RT	GTTGGCTCTGGTGCAGGGTCCGAGGTATTCGCACCAGAGCCAAC ACACGT	
	Forward	TTGGTGGAGAAGCAGGGC	
	Universal reverse	GTGCAGGGTCCGAGGT	80 pb

Musa* target genes of miRNAs, primer sequences and supporting information for RT-qPCR analysis of differential gene expression during interaction with *Pseudocercopsora musae				
**Target reference *Musa acuminata* DH Pahang Gene ID**	**Predicted gene function**	**Forward Primer (5'-3')**	**Reverse Primer (5'-3')**	**Expected Amplicon size (bp)**
Ma03_g12850	pentatricopeptide repeat-containing protein-like	TACTGGGTGTTGCTGTCTAATG	CCAACCTCAACCCAGCTAAA	120 pb
Ma04_g10650	Acireductone dioxygenase	GACACAAGCAGAGTGTCTTCTA	TGGTCCTCATCACTCTCATCTA	102 pb
Ma06_g18000	transport inhibitor response 1-like protein	TGTTTGTTGGCAACTGCTATG	CTTGACGCTCAGCGAATTTATC	96 pb
Ma06_g38750	scarecrow-like protein 6	GACACAAGCAGAGTGTCTTCTA	AGAGGGAAAGGGATGTTTGG	100 pb
Ma08_g07960	30S ribosomal protein S17	TAGCAGCAGCATCAGTTCTC	GAGGAGAGTTGGAGGAAAGAAG	92 pb
Ma09_g28300	squamosa promoter-binding-like protein 14	GTCTCTCTTACTCTCTCCAAAGAAG	GCAGCTCTCTCTACAGCTTATC	89 pb
Ma11_g01530	auxin response factor 12-like	CAGACACATTCCAGGTTCCTAA	GAAGTCCGGCTTGTCGTAAA	100 pb
Ma11_g24070	probable alpha-glucosidase	CACTCTCGATCCCATCAACTTC	CATACTTCTGCCCATTCTCGT	105 pb
